# Anxiety Level and Cardiac Autonomic Modulations in Coronary Artery Disease and Cardiac Syndrome X Patients

**DOI:** 10.1371/journal.pone.0170086

**Published:** 2017-01-09

**Authors:** Mohamed Faisal Lutfi

**Affiliations:** Department of Physiology - Faculty of Medicine and Health Sciences - Al-Neelain University, Khartoum, Sudan; University of Bologna, ITALY

## Abstract

**Background:**

Anxiety and cardiac autonomic modulations (CAM) were thoroughly investigated in coronary artery disease (CAD) and cardiac syndrome X (CSX) patients worldwide, but not among Sudanese with similar pathology.

**Aims:**

To compare levels of anxiety and CAM between Sudanese patients with CSX and CAD.

**Materials and Methods:**

Anxiety was evaluated in 51 CAD and 26 CSX patients using Taylor Manifest anxiety score (TMAS) questionnaire while heart rate variability derived indices were used to assess CAM, namely natural logarithm of low frequency (LnLF), high frequency (LnHF) and LF/HF ratio (LnLF/HF).

**Results:**

Low anxiety levels were achieved by 6 (23.1%) and 9 (17.6%) patients with CSX and CAD respectively. High anxiety level was achieved by only one (3.8%) patient, who was suffering from CSX. TMAS was significantly higher in CSX (31.27 (21.97)) compared to CAD (21.86 (12.97), P = 0.021). However, abnormally increased anxiety was not associated with higher risk of CSX. LnLF, LnHF and LnLF/HF were comparable in CAD and CSX patients.

**Conclusion:**

CSX and CAD patients showed comparable CAM. Although anxiety levels were higher in CSX compared to CAD, TMAS ≥ 35 failed to show significant association with CSX.

## Introduction

Presence of typical angina and significant risk factors of atherosclerosis usually points to coronary artery disease (CAD) [1]. However, 10% to 40% of patients with typical angina may have normal coronary angiograms [2, 3]. Angina in such conditions may be attributed to microvascular ischemia [4] and/or augmented sensitivity to pain [5], which are the suggested etiologies for cardiac syndrome X (CSX). CSX is defined by the presence of the triad: typical angina, positive cardiac stress test(s) and normal coronary angiography [3]. Both CSX [6] and CAD [7, 8] express features of metabolic syndrome, which compromise differentiation of these diseases in the clinical settings. Anxiety and other psychological stresses are among the risk factors of CAD [9] especially in younger adults [10]. Likewise, same psychological stresses may provoke cardiac pain in patients with CSX [3, 11]. In both conditions, namely CSX and CAD, anxiety can reset cardiac autonomic modulations (CAM) towards predominance of sympathetic over the vagal tone, putting victims at higher risk of arrhythmia and sudden death [12–14].

Recent studies on cardiac autonomic modulations (CAM) are usually dependent on the evaluation of the chronotropic effects of sympathetic and parasympathetic fibers on the heart [15, 16]. The frequency domain method used for evaluation of heart rate variability (HRV) is among the most useful indicators of CAM [16]. In the frequency domain method, the popular indices used to determine sympathetic and parasympathetic CAM are low frequency (LF) and high frequency (HF) of power spectrum density, respectively [15]. Alternatively, low frequency/high frequency ratio (LF/LH) is used to evaluate sympathovagal balance [15, 16].

In Sudan, the diagnostic yield of coronary catheterization and risk factors of abnormal coronary angiograms in patients with typical angina are poorly understood and remains to be explored by extensive researches [17–20]. Although anxiety and CAM are well studied in candidates of coronary catheterization worldwide [12, 13, 21, 22], nothing is known about these parameters in Sudanese patients with the same pathology. This study aimed to compare levels of anxiety and CAM in Sudanese patients with CSX and CAD.

## Materials and Methods

Ethical issues of the study were cleared from the ethics review committee (ERC)—Faculty of Medicine—Khartoum University—Sudan. All volunteers signed a written informed consent before they joined the study.

Seventy seven patients with a history of typical angina were seen in Al-Shaab cardiac center, Khartoum, Sudan, in the morning of the same day intended for diagnostic coronary catheterization (DCC). Following evaluation of past medical history and clinical examination, the patients were allowed to fill Taylor Manifest anxiety score (TMAS) questionnaire [23]. TMAS is a simple thirty-five items questionnaire, which was self-completed by patients. Each item is scored from zero (not at all) to four (very much so). After filling the questionnaire, the scores were added to give a final score with a minimum of zero and a maximum of 140. Subjects who scored 34 or less were considered non-anxious while those who fell in the ranges of 35–70 and 71–140 were considered as suffering from low and high anxiety respectively [23]. Body mass index (BMI) and mean arterial blood pressure (MABP) of the studied groups were calculated by the formulae:

BMI (kg/m^2)  = weight (kg)/(height (m^2)

$$\text{MABP }\left(\text{mmHg}\right)\;=\text{ Diastolic blood pressure+}\frac{1}{3}\text{ Pulse pressure}$$

Where,

Pulse pressure  = Diastolic blood pressure−Systolic blood pressure

Fifty one of the studied patients where proved to suffer from narrowing of half (or more) of the caliber of one (or more) of the major coronary artery/arteries and were considered as CAD [24]. The remaining studied patients (N = 26) were diagnosed as CSX according to what was described by Crea and Lanza [25] i.e. typical angina, significant ST segment depression in ECG performed at rest or during stressful conditions and normal coronary angiogram. A Bluetooth ECG transmitter and receiver (DM systems (Beijing) Co. limited—China) was used for electrocardiographic (ECG) recording and subsequent evaluation of CAM based on HRV-derived values. ECG was recorded for each studied subject in the supine position for 5 minutes after ensuring absence of artifacts on the ECG screen and comfortable breathing. Natural logarithms were used to express low frequency (LnLF) and high frequency (LnHF) powers of frequency domain HRV.

Natural logarithm of LF/HF (LnLF/HF) was used to evaluate sympathovagal balance among the studied groups [15, 16]. Bluetooth ECG transmitter and receiver software also gives the average heart rate (HR) during the period of ECG recording (5 minutes).

Statistical package for the social sciences (SPSS) for windows, version 16.0 (SPSS Inc., Chicago, IL, USA) was used for Statistical evaluation. Studied variables were described with means (M), standard deviations (SD) and bar charts showing M±SD. Proportions of the studied groups were expressed in percentages (%) and 95% confidence intervals (CI). Unpaired T-test was used to assess differences in the means of the studied variables between CSX and CAD patients. General linear model was used to adjust for gender and HR as possible cofounders while comparing CAM between CSX and CAD patients. Univariate analysis was carried out to evaluate the association between CAD and gender, past history of hypertension, diabetes mellitus, smoking and anxiety (TMAS ≥ 35). Results of univariate analyses were expressed by odds ratios (OR), which presently described the ratio of the odds of an event occurring in patients with CAD to the odds of the same event occurring in subject with CSX. In the ECG, HR is inversely proportional to RR intervals, from which all frequency domain HRV measures are derived. Consequently, partial correlations were used to adjust for the variations in HR of patients while testing for significant associations between indicators of CAM and TMAS. P < 0.05 was considered significant.

## Results

Coronary artery catheterization of the studied subjects (N = 77) revealed 51 (66.3%, 95% CI = 55.1%–75.8%) patients with CAD and 26 (33.7%, 95% CI = 24.2%–44.9%) subjects with CSX. Age of CAD patient (56.75 (8.46) years) was higher, while BMI (26.91 (4.04) Kg/m^2^) was lower, compared with CSX (49.54 (7.92) years, P = 0.001 and 29.88 (4.95) Kg/m^2^, P = 0.008 respectively), Table 1. Male gender was predominant in CAD patients, Table 1.

**Table 1:** Distribution of gender, age, BMI, MABP and HR among the studied groups.

	CSX	CAD	P
Male gender, N (%)	15 (57.7%)	44 (86.3%)	0.005
Age (Years), M (SD)	49.54 (7.92)	56.75 (8.46)	0.001
BMI (kg/m2), M (SD)	29.88 (4.95)	26.91 (4.04)	0.008
MABP (mmHg), M (SD)	99.03 (14.66)	93.71 (13.09)	0.134
HR (BPM), M (SD)	65.17 (8.64)	72.19 (12.59)	0.054

Males (OR = 4.61, P = 0.001) and diabetic patients (OR = 5.29, P = 0.006) have higher risk to develop CAD compared to CSX, Table 2.

In contrast, MABP, HR, past history of hypertension and smoking were not significantly different between CAD and CSX patients, Tables 1 and 2.

**Table 2:** Association between male gender, hypertension, diabetes mellitus, smoking and anxiety and CAD.

	OR	95% CI	P
Male Gender	4.61	1.51–14.05	0.007
Hypertension	1.31	0.51–3.39	0.577
Diabetes Mellitus	5.29	1.60–17.53	0.006
Smoking	1.56	0.59–4.13	0.368
Anxiety (TMAS ≥ 35)	0.53	0.19–1.49	0.229

**Fig 1:**
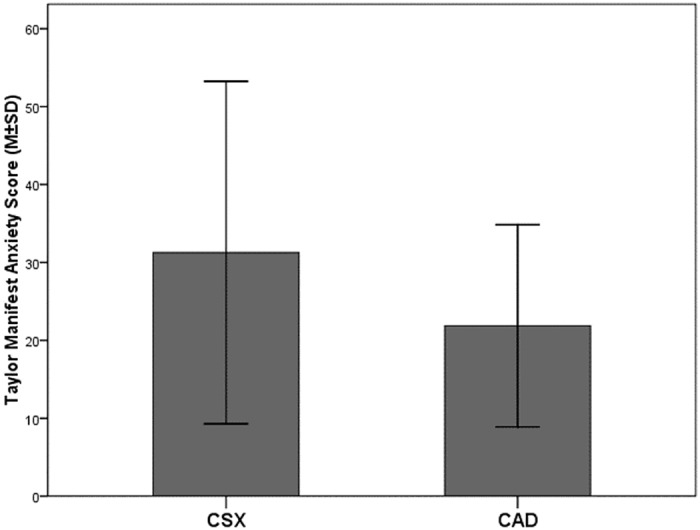
Distribution of Taylor-Manifest anxiety scores among CSX and CAD patients.

TMAS was significantly higher in CSX (31.27 (21.97)) compared to CAD (21.86 (12.97), P = 0.021), Fig 1.

Normal anxiety levels were achieved by 19 (73.1%, 95% CI = 53.9%–86.3%) and 42 (82.4%, 95% CI = 69.8%–90.4%) patients with CSX and CAD respectively. In contrast, 6 (23.1, 95% CI = 11.0%–42.1%) and 9 (17.6%, 95% CI = 9.6%–30.3%) patients in the respective groups achieved low anxiety levels. High anxiety level was achieved by only one (3.8%, 95% CI = 0.7%–18.9%) patient, who was suffering from CSX. Abnormally increased anxiety (as indicated by TMAS ≥ 35) was not associated with higher risk of CSX, Table 2.

LnLF, LnHF, and LnLF/HF were comparable in CAD and CSX patients, Table 3.

**Table 3:** Correlations between TMAS and indicators of cardiac autonomic modulations in CSX and CAD patients.

	CSX	CAD	P	
	M (SD)	M (SD)	Non-Adjusted	Adjusted for HR and gender
LnLF	4.92 (1.26)	4.54 (1.44)	0.412	0.464
LnHF	4.87 (1.41)	4.42 (1.44)	0.369	0.647
LF/HF	1.59 (1.38)	1.84 (1.73)	0.636	0.981

None of CAM parameters showed significant correlation with TMAS, Table 4.

**Table 4:** Correlations between TMAS and indicators of cardiac autonomic modulations in CSX and CAD patients.

	CSX				CAD			
	Non-Adjusted		Adjusted for HR and gender		Non-Adjusted		Adjusted for HR and gender	
	*r*	P	*r*	P	*r*	P	*r*	P
LnLF	0.26	0.411	0.30	0.400	-0.04	0.834	0.03	0.877
LnHF	-0.09	0.785	-0.06	0.862	-0.11	0.600	0.17	0.447
LnLF/HF	0.42	0.173	0.21	0.563	0.09	0.656	0.13	0.540

## Discussion

According to the present results, two third of the patients undergoing coronary catheterization were suffering from CAD. The risk of CAD increased with age, male gender, lower BMI and presence of diabetes mellitus. In contrast, presence of past history of hypertension, smoking and anxiety did not increase the odds of having CAD in the studied subjects. Although anxiety levels were higher in CSX compared to CAD, TMAS ≥ 35 failed to show significant association with CSX.

Previous studies showed that up to 40% of patients with angina have normal coronary angiograms [2, 3], and the diagnosis of abnormal coronary arteries is five times more common in men than women [26]. Alternatively, patients with typical angina and normal or non-obstructive coronary angiograms are predominantly females [27]. Soler et al showed higher risk factors of CAD among elderly individuals [28]; however, other investigators demonstrate stronger risk factors associations among younger individuals compared to older women and men [29]. Although increased BMI is a known risk for CAD [30, 31], our results demonstrated higher BMI among CSX. This finding can be explained if diabetes mellitus among CAD is insulin dependent [32] and by predominance of male gender among the same group. Failure of the present results to demonstrate higher blood pressures in CAD do not necessarily disagree with previous reports [33], but could simply reflect the effects of already established antihypertensive treatments in those with high blood pressure readings.

Anxiety is proved to be associated with CAD [9]. However, most studies in this field targeted middle-age or older patients and therefore the observed association between anxiety and CAD may be explained by the fact that both diseases are age related [10]. Investigations of the long-term cardiac effects of psychological disturbances on Swedish men showed anxiety as an independent predictor of subsequent CAD events in younger age group [34]. In our study, TMASs of subjects with CSX were significantly higher compared to patients with CAD. This finding should not be interpreted contrary to current understanding of anxiety as a risk factor for CAD. Although patients who showed higher TMAS score were proved to have normal coronary arteries, they experienced typical angina attacks and hence are difficult to be considered as normal. Actually, non-cardiac, non-esophageal chest pain was extensively studied in the nineties of the last century and was mostly attributed to anxiety and other psychological factors [35, 36]. More recent reports on chest pain and normal angiography suggest the possibility of CSX whose main potential pathogenic mechanisms are related to anxiety [37]. A pilot study conducted on patients with typical chest pain and completely normal coronary angiograms revealed that CSX patients with high trait anxiety are at risk of having more microvascular ischemia of the heart [38]. In a more recent report, patients with CSX have higher prevalence of anxiety and other psychiatric comorbidities and they do benefit from psychological support [11].

CAM was extensively studied in patients with typical chest pain. Nearly all previous reports in this field compared patients with either CAD or cardiac syndrome X to healthy control subjects [12–14, 39, 40]. To our knowledge, the present study is probably the first report that explores the possible CAM differences between patients with CSX and CAD. The results of the present study reveal no differences in the indicators of sympathovagal balance, namely, LnLF, LnHF, and LnLF/HF. Using 24-hour ECG monitoring in patients CSX, Ponikowski et al observed CAM during attacks of ST-segment depression [40]. LF and HF bands were measured in the 30 minutes preceding the onset of transient ischemia. Results showed that ST-segment depression associated with tachycardia was preceded by low HF and high LF/HF ratio, but not in attacks of ST-segment depression associated with unchanged heart rate. Ponikowski et al concluded that CSX is characterized by sympathetic predominance during attacks of ST-segment depression and tachycardia, probably due to vagal tone withdrawal. Two years later, Lee et al [39] were able to give further support for Ponikowski et al implications when they demonstrated progressive shortenings and lengthening of RR intervals before and after myocardial ischemic episodes in patients with CSX. HF was significantly reduced whereas LF band did not change before ischemic episodes. Lee et al therefore confirm vagal withdrawal during ischemic attacks in patients with CSX. In another study, Frobert et al [41] compared HRV parameters in healthy subjects to CSX patients with either positive or negative stress ECG tests. Results revealed evidence of vagal withdrawal in CSX patients with negative stress ECG test. In contrast, there was no change in CAM of those with positive stress ECG test. Frobert et al findings gave imperative cues for heterogeneity of patients with CSX. Accordingly, it seems logical that different patterns of CAM characterize different subclasses of CSX patients. Whether CAM of CSX subjects will appear different from another group of patients depend on which subclass of CSX subjects predominate in the first group. This assumption may partially explain why our results failed to show significant differences in CAM between CSX and CAD patients. Alternatively, vagal withdrawal in both CSX and CAD [12–14, 39–41] may be the other explanation for comparable CAM in our studied groups.

With only few exceptions [42], previous reports repeatedly showed an inverse relationship between anxiety level and vagal tone [43, 44]. Based on measures of CAM, the level of anxiety is expected to correlate inversely with HF and proportionally with LF and LF/HF [45]. Failure of our results to demonstrate the expected trend between TMAS and measures of CAM may partly be explained by the relatively low anxiety levels in the studied groups. Further researches are required to explain the, probably hidden, correlations between anxiety measures and CAM in CSX and CAD patients we studied.

## Conclusion

The results of the present study showed no differences in the sympathovagal balance between CSX and CAD, probably because of vagal withdrawal in both groups. Although anxiety levels were higher in CSX compared with CAD, TMAS ≥ 35 failed to show significant association with CSX. The relatively decreased anxiety levels in CSX and CAD may explain absence of significant correlations between TMAS and indictors CAM in the studied groups.
